# Insights into the Microbial and Viral Dynamics of a Coastal Downwelling-Upwelling Transition

**DOI:** 10.1371/journal.pone.0137090

**Published:** 2015-09-01

**Authors:** Gustavo Bueno Gregoracci, Ana Carolina dos Santos Soares, Milene Dias Miranda, Ricardo Coutinho, Fabiano L. Thompson

**Affiliations:** 1 Departamento de Ciências do Mar, UNIFESP Baixada Santista. Santos, SP, Brazil; 2 Instituto de Biologia, Universidade Federal do Rio de Janeiro (UFRJ), Rio de Janeiro, RJ, Brazil; 3 Laboratório de Vírus Respiratórios e do Sarampo, Instituto Oswaldo Cruz (IOC)-FIOCRUZ, Rio de Janeiro, RJ, Brazil; 4 Instituto de Estudos do Mar Almirante Paulo Moreira (IEAPM), Arraial do Cabo, RJ, Brazil; 5 Laboratório de Sistemas Avançados de Gestão de Produção—SAGE—COPPE, Centro de Gestão Tecnológica—CT2, UFRJ, Rio de Janeiro, RJ, Brazil; CAS, CHINA

## Abstract

Although previous studies have described opposing states in upwelling regions, i.e., the rise of cold nutrient-rich waters and prevalence of surface warm nutrient-poor waters, few have addressed the transition from one state to the other. This study aimed to describe the microbial and viral structure during this transition and was able to obtain the taxonomic and metabolic compositions as well as physical-chemical data. This integrated approach allowed for a better understanding of the dynamics of the downwelling upwelling transition, suggesting that a wealth of metabolic processes and ecological interactions are occurring in the minute fractions of the plankton (femto, pico, nano). These processes and interactions included evidence of microbial predominance during downwelling (with nitrogen recycling and aerobic anoxygenic photosynthesis), different viral predation pressures over primary production in different states (cyanobacteria vs eukaryotes), and a predominance of diatoms and selected bacterial and archaeal groups during upwelling (with the occurrence of a wealth of nitrogen metabolism involving ammonia). Thus, the results provided insights into which microbes, viruses and microbial-mediated processes are probably important in the functioning of upwelling systems.

## Introduction

Upwelling regions sustain a high productivity of primary producers, such as diatoms and fish. Although the Brazilian coast is not among the five major upwelling coasts of the world, namely, the Canary current system off the Iberian Peninsula and northwestern Africa, the Benguela current system off southwestern Africa, the Peru current system off western South America, and the western coast of the United States [1, 2], the southeastern portion of the coast includes the South Brazil Bight, a high productivity zone characterized by upwelling events [3]. Within this large area, extending from Cabo Frio (23°S 42°W) to Cabo Santa Marta (28.5°S 48.6°W), the northernmost portion has received more attention and study [2, 3].The Cabo Frio region represents a “turning” point in the Brazilian coast, where the coastline changes from north-south to east-west, and is thus subjected to the strongest wind driven upwelling events of any location on this coast [2]. NE-E winds from the South Atlantic anticyclone transport surface water away from the coast, causing water from the South Atlantic Central Water (SACW) current to rise, particularly during austral spring and summer seasons [2–4]. The passage of southern (S-SW) cold fronts interrupts the upwelling, and surface waters from the Continental Water and the Brazilian Current (Tropical Water) prevail [2–4].

Longer upwelling events, particularly in the austral spring, allowed the distinction of three phases: 1) an upwelling of cold and nutrient-rich waters; 2) a phytoplankton production phase in which nutrients are incorporated as biomass; and 3) a downwelling phase in which phytoplankton and nutrient concentration decline [4]. In austral summer, the more frequent passage of cold fronts provides shorter and weaker upwelling intermixed with frequent downwelling. Although the distinction of different phases has been clearly shown in previous studies [2, 4, 5], few studies have focused on the transition from one state to the other [6], and to our knowledge, none describe the temporal effect of the transition on the microbial community. A common approach in the study of upwelling events is the description of organisms during upwelling occurrences [4] or in contrasting conditions of upwelling and downwelling [5, 7–10], while the dynamics of a transition between these extremes is overlooked by most (but see [6]). The aim of this study was the description of how the passage of a summer cold front affects the viral and microbial communities, which was achieved through an integrated approach involving metagenomic analysis via high throughput sequencing as well as measurements of microbial abundance and water chemistry analysis.

## Material and Methods

### Sampling site and dates

Sub-surface (~0.5 m deep) water samples were collected before noon each day during January 18^th^-20^th^ 2012 using a speedboat to reach the location (upwelling field in Arraial do Cabo, Rio de Janeiro, Brazil; lat -22.995336, long -42.016656). These days were thought to represent the 1^st^ to 3^rd^ days of a summer upwelling event, the occurrence of which was confirmed prior to sampling by the low water temperature (below 18°C) measured using a dive computer and after sampling by wind direction data kindly provided by the Meteorological Data Storage Section (SADMET) of the Brazilian National Meteorological Institute (INMET). The use of temperature as proxy for upwelling occurrence is supported by previous studies [2, 4, 7, 10] and the personal experience of one of the authors (RC). No specific permissions were required for water sampling.

### Water sampling and field processing

Approximately 100 L of seawater was collected each day using a manual pump and transported within an hour to the field base at Instituto de Estudos Avançados Paulo Moreira in Arraial do Cabo, RJ, Brazil, for further processing and filtration. Flow cytometry samples were fixed and frozen in liquid nitrogen. Water samples for chemical analysis were filtered (chlorophyll-a) and frozen (all). Metagenomic samples were pre-filtered (100 μm), and tangentially filtered (200 kDa) to a final volume of ~0.5 L from approximately 40–60 L. The concentrate was then syringe-filtered through 0.22 μm Sterivex filters to separate cells and viruses. The filters were frozen with SET buffer, while the cell-free viral concentrate was refrigerated, protected from light.

### Chemical Analysis

All environmental parameters were analyzed by standard oceanographic methods [11, 12]. At least three replicates were analyzed per parameter per day. Temperature and salinity were evaluated using CTD or salinity meters from YSI. Chlorophyll *a* analyses were performed after the vacuum filtration (<25 cm of Hg) of 2 L of water. The filters (Whatman GF/F glass fiber) were extracted overnight in 90% acetone at 4°C and analyzed by spectrophotometry or fluorimetry. Inorganic nutrients were also analyzed by the following methods [11]: 1) ammonia by indophenol, 2) nitrite by diazotization, 3) nitrate by reduction in a Cd-Cu column followed by diazotization, 4) total nitrogen by digestion with potassium persulfate following nitrate determination, 5) orthophosphate by reaction with ascorbic acid, 6) total phosphorous by acid digestion to phosphate, and 7) silicate by reaction with molybdate.

### Flow cytometry counts

Different fixatives were used for different samples, each of which was performed separately and in triplicate: 0.55% glutaraldehyde for viruses, 1% paraformaldehyde for autotrophic eukaryotes and a 1% paraformaldehyde/0.05% glutaraldehyde mixture for bacteria and archaea. An abundance of both viruses and microbes was determined from two replicate samples of seawater by flow cytometry with Syto-13 (Life Technologies, Carlsbad, CA) with minor modifications [13]. Primary producers were detected by auto-fluorescence by flow cytometry. Microbial cells with high (HNA) and low (LNA) nucleic acid contents were differentiated and quantified by the fluorescence intensity of Syto-13 during flow cytometry.

### Laboratory processing and DNA extraction—Viruses

Viral concentrates were subjected to further concentration by ultracentrifugation at 85,000 g and 4°C for 7–14 h to a final volume of less than 10 ml. Each daily sample was processed separately, and pooling was performed only between aliquots of the same sample. After ultracentrifugation, the pellets were slowly resuspended for at least 24 h, under refrigeration and protection from light, in a fraction of their own supernatant. The ultra-concentrates were then extracted by a proteinase K-SDS lysis, phenol-chloroform extraction, and ethanol precipitation. Quality control was achieved using Nanodrop absorbance. Contamination was checked with PCR reactions for *rRNA 16S* and *cox1* genes using a complex metagenome containing all types of microbial cells as positive control [14]. Positive reactions implied contamination, in which case the aliquots were discarded. DNA quantification was performed using Bio-analyzer and Qubit; no DNA amplification (MDA) was necessary as the protocol provided enough viral material (a few μg of DNA per sample) for direct preparation.

### DNA extraction–Cells

The cells in Sterivex filters were extracted as previously described [15], and each daily sample was processed separately; only aliquots of the same sample were pooled. Briefly, they were treated with lysozyme and lysed with proteinase K-SDS inside the filters. After incubation, the supernatant was removed, extracted with phenol-chloroform-isoamyl alcohol, and precipitated with ethanol-ammonium acetate. Quality control was performed using Nanodrop absorbance and quantification using Bio-analyzer and Qubit.

### Ion Sequencing

Cellular DNA was fragmented using water-bath sonication (Bioruptor) according to the manufacturer’s protocol: five 15-minute cycles at low power, with half a minute on and half a minute off. The viral DNA, which is more fragile, was subjected to an alternative protocol: one 15-minute cycle only at low power, with 20 seconds on and half a minute off. Regarding the fragmentation results, both the distribution and quantitation were checked in a Bio-analyzer. Both viral and cellular sheared DNA were then used for library preparation using an Ion Plus Fragment Library kit. Libraries were emulsion PCR amplified using the Ion One Touch 200 Template Library kit v2 DL and sequenced using the Ion PGM 200 Sequencing kit. Q20 data were separated using the dedicated Torrent Server as fastq files.

### Quality control and data analysis

Sequences were automatically annotated using MG-Rast [16]. Sequences were only dereplicated because emulsion PCR is also involved in the Ion Torrent protocol. Reads were annotated on a “best hit” basis against GenBank for taxonomy and Subsystems for functions, both with an e-value cutoff of 10^−5^. Because GenBank also includes Viral RefSeq data, no specific viral pipeline was used. Viral reads were separated using the Workbench tool prior to functional annotation. As MG-Rast multiplies hits for functions involved in several subsystems, a homemade Perl script was used for manual curation of the Functional annotation (available upon request). Briefly, this script identifies multiplied functions and either splits the abundance between different functions or allows the manual elimination of misplaced functions, as chosen by the user. Samples were then compared pairwise in Stamp [17] using a two sided hybrid G-test (with Yates continuity correction) and Fisher’s exact test, with the confidence interval calculated by the asymptotic method with continuity correction and multiple test correction by the Benjamin-Hochberg false discovery rate method. Corrected p-values were considered statistically significant if less than 0.05. All metagenomic data is publicly available in the MG-Rast website, where it is deposited as part of the “Upwelling Arraial do Cabo” project (http://metagenomics.anl.gov/linkin.cgi?project=2518), or alternatively it is available in BAMBA (Meirelles et al. in press), under project identifier (https://marinebiodiversity.lncc.br/metacatui/#view/pmeirelles.17.1).”

## Results

### Physical chemical analysis and microbial abundance

The water temperature measured during sampling and wind direction data (S1 Fig) confirmed the occurrence of an upwelling event, as stated in the Methods (Sampling site and dates). However, the wind direction showed the passage of a cold front during January 16^th^, with the 17^th^ being a transition period. A marked decrease in ammonia from the 18^th^ to the 19^th^-20^th^ was observed, as were decreases in chlorophyll and pheophytin in the microbial fraction smaller than 100 μm (Table 1). As the upwelling progressed, there was a marked increase in silicate on the 20^th^, with accompanying increases in phosphorus (both orthophosphate and total) and nitrate, particularly from the 18^th^ to the 19^th^-20^th^ (Table 1). The bacterial, archaeal, synechococcal, picoeukaryotic, nanoeukaryotic and viral abundances decreased slightly from the 18^th^ to the 20^th^, although the viral fractions (V1—V3) responded differently to the upwelling transition (Table 2).

**Table 1:** Physical-chemical variables.

	Sampling date (2012)		
Chemical variable^a^	Jan 18th	Jan 19th	Jan 20th
Chlorophyll-a (μM)	1.11 ± 0.13	0.69 ± 0.06	0.49 ± 0.05
Phaeophytin-a (μM)	0.85 ± 0.12	0.47 ± 0.03	0.43 ± 0.05
Silicate (μM)	3.93 ± 0.09	5.14 ± 0.01	16.87 ± 0.06
Water temperature (°C)	17	17	16
Salinity	35.23 ± 0.03	36.36 ± 0.04	35.61 ± 0.02
Orthophosphate (μM)	0.6 ± 0.01	0.86 ± 0.01	0.88 ± 0.01
Total phosphorus (μM)	0.84 ± 0.08	0.94 ± 0.04	0.95 ± 0.04
Ammonia NH_3_ (μM)	5.44 ± 9.15	0.26 ± 0.03	0.25 ± 0.06
Nitrite NO_2_ (μM)	0.3 ± 0.01	0.27 ± 0.01	0.23 ± 0.00
Nitrate NO_3_ (μM)	4.27 ± 0.20	7.17 ± 0.57	7.73 ± 0.17
Total Nitrogen (μM)	18.58 ± 1.94	46.25 ± 0.11	39.32 ± 1.16

^a^ area averages of three replicates with standard deviation

**Table 2:** Microbial and viral abundance.

	Sampling date (2012)								
Cytometry counts^1^	Jan 18th			Jan 19th			Jan 20th		
Prokaryotes (cells/ml)	1.95E+06	±	4.22E+04	1.04E+06	±	4.40E+04	7.89E+05	±	4.27E+04
HNA (cells/ml)	1.41E+06	±	2.90E+04	7.12E+05	±	2.85E+04	5.21E+05	±	2.99E+04
LNA (cells/ml)	5.40E+05	±	1.33E+04	3.27E+05	±	1.90E+04	2.68E+05	±	1.28E+04
% HNA	72.36	±	0.09	68.56	±	0.87	65.98	±	0.24
% LNA	27.64	±	0.09	31.44	±	0.87	34.02	±	0.24
HNA / LNA	2.62	±	0.01	2.18	±	0.09	1.94	±	0.02
Synechococcus (cells/ml)	1.85E+04	±	4.85E+02	7.82E+03	±	4.68E+02	4.74E+03	±	4.85E+02
Picoeukaryotes (cells/ml)	1.20E+04	±	1.41E+02	6.11E+03	±	4.51E+01	5.15E+03	±	8.63E+01
Nanoeukaryotes (cells/ml)	2.16E+03	±	1.25E+02	1.01E+03	±	4.29E+01	8.76E+02	±	1.01E+02
Viruses (viral particles/ml)	8.41E+06	±	3.68E+05	7.26E+06	±	2.64E+05	4.88E+06	±	7.31E+05
Microbes/virus ratio	0.23			0.14			0.16		
% V1	57.80	±	0.99	59.09	±	0.69	56.89	±	2.83
% V2	30.12	±	0.86	30.14	±	0.44	32.23	±	2.71
% V3	12.07	±	0.14	10.77	±	0.26	10.88	±	0.14

^1^ averages of three replicates plus standard deviation

### Metagenomic samples

All samples, including viral DNA, resulted in good DNA yields on the order of micrograms, precluding the need for amplification. Our data also showed an improvement resulting from our modification of the viral protocol. While popular protocols [18] use a cesium chloride step to concentrate only a fraction of the total viral ultrafiltrate after TFF, our methodology involved the concentration of all available material to avoid the biases introduced by cesium chloride. Additional losses by several different chemical extractions in tandem were also avoided [18], and altogether higher yields were obtained (few micrograms of DNA), precluding the need for non-specific amplification, which is known to introduce bias [19, 20]. Neither PCR controls (*16S rRNA* and *cox1*) nor flow cytometry analyses of the first few samples (data not shown) revealed any cellular contamination in this protocol, corroborating the extraction of viral only DNA. A few aliquots did amplify small amounts of *cox1*, probably from handler manipulation during extraction, and they were discarded. The quality of our data seems at least as good as that of similar virome data from the Pacific Ocean Virome [21] when POV viromes were annotated with MG-Rast (data not shown but deposited along this project).

S1 Table summarizes the sequencing technical data. Nearly 900 Mb of viral sequences and nearly 600 Mb of microbial sequences were generated, totaling nearly 7 million reads and clearly demonstrating the improvement achieved using this modified viral DNA extraction and library preparation protocols. Viral reads subjected to this tailored protocol additionally produced longer reads (viral averages 255–268 bp vs cellular averages 162–191 bp). Bacteria and archaea comprised the majority of cellular reads identified in the cellular metagenome, although eukaryal identification increased over the sampling period (S1 Table). Functions could be attributed to 30–55% of these reads. Viral metagenomes yielded far fewer sequences that were identifiable taxonomically (2.5–6.5%) and functionally (0.64–2.2%) (S1 Table). It is important to note that viral annotation was filtered taxonomically and functionally using MG-Rast and the homemade Perl script described previously, which considerably lowered the abundances in exchange for improved confidence in the annotations.

### Metagenomic characterization of viruses

**Fig 1:**
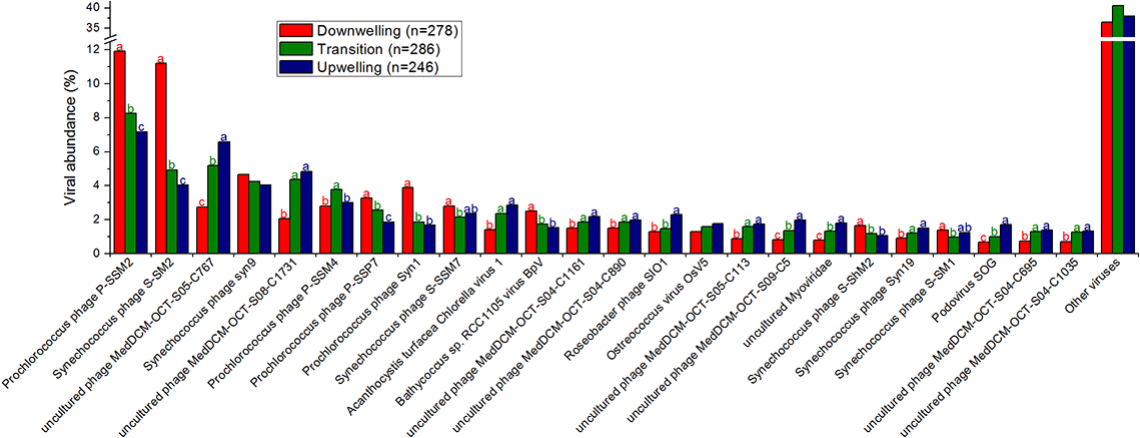
Most abundant viral species–over 1% average abundance (relative percentage). The remaining viruses are grouped as “Other viruses”. The total number of reads included in this analysis is 50,164. Different letters (a > b > c) represent statistically significant differences (corrected p <0.05), while ab are intermediate values.

**Fig 2:**
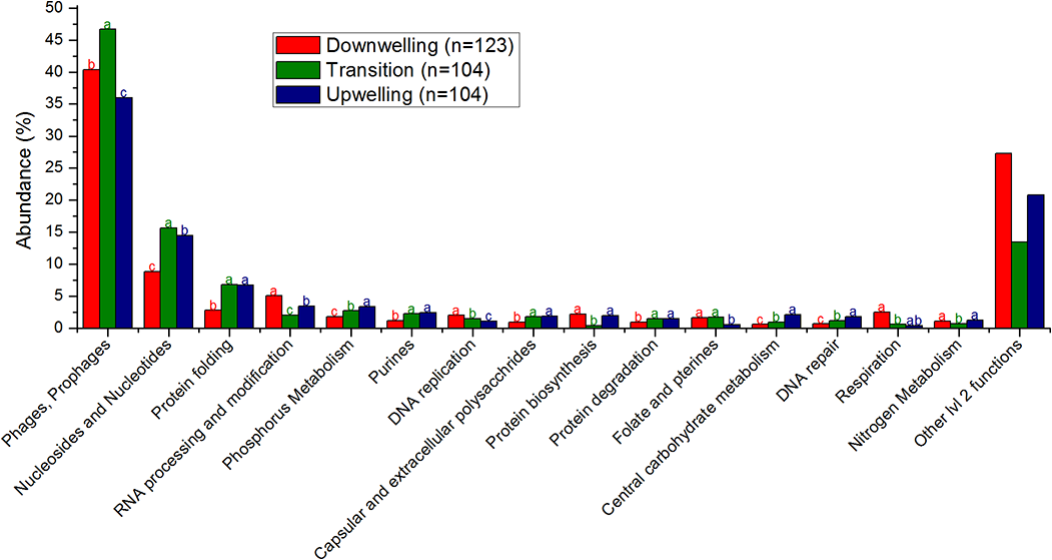
Significantly different most abundant viral functions and auxiliary metabolic genes (Subsystems lvl2)–over 1% average abundance (relative percentage). The remaining functions are grouped as”Other lvl2 functions”. The total number of reads included in this analysis is 47,833. Different letters (a > b > c) represent statistically significant differences (corrected p <0.05), while ab are intermediate values.

Most recognizable viruses were cyanobacterial phages, algal viruses or similar from deep chlorophyll maximum phages from other studies (Fig 1). Among the most abundant phages, it was also possible to locate a *Roseobacter* infecting species. As the upwelling progressed, the abundance of most cyanobacterial phages decreased, as did the overall richness of phage species (Fig 1). By contrast, most of the phages similar to those found on a deep chlorophyll maximum (DCM, see Discussion) increased in abundance with the upwelling, as did the *Roseobacter* phages and *Acanthocystis chlorella* viruses. Among the most abundant viruses, only a few strains responded directly to the transition stage, either positively or negatively, and most belong to cyanophages, DCM phages or heterotrophic *Gammaproteobacteria*, such as T4, P22 and nt-1. S2 Table presents the detailed taxonomical annotation and statistical analysis, but all aspects highlighted in the text refer to statistically significant observations. Most recognizable functions were related to viral functions, such as phages, prophages (structural components), nucleotides synthesis, nucleic acids processing and replication (Fig 2, S3 Table). Some abundant functions, however, seemed to be auxiliary metabolic genes, being annotated as, e.g., respiration (dehydrogenases), phosphorus metabolism and uptake, and nitrogen metabolism (ammonia assimilation and nitrogen fixation). Interestingly, over a third of viral functions presented a reduced abundance during the transition (19^th^) relative to the downwelling (18^th^) or upwelling (20^th^) conditions. Notably, basic viral functions (phages, prophages, capsid proteins, nucleosides and nucleotides metabolism) were increased during the transition, while most remainder accessory functions were reduced, including tRNA and ribosome modification proteins. S3 Table presents the detailed functional annotation and statistical analysis, but all aspects highlighted in the text refer to statistically significant observations.

### Metagenomic characterization of microbial cells

**Fig 3:**
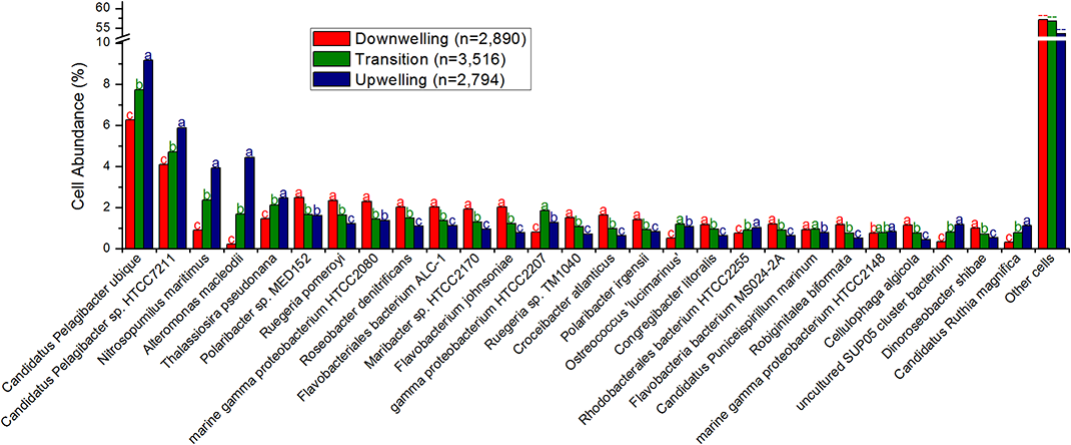
Most abundant cellular species genes–over 0.75% average abundance (relative percentage). The remaining cells are grouped as “Other cells”. The total number of reads included in this analysis is 1,528,681. Different letters (a > b > c) represent statistically significant differences (corrected p <0.05), while ab are intermediate values.

**Fig 4:**
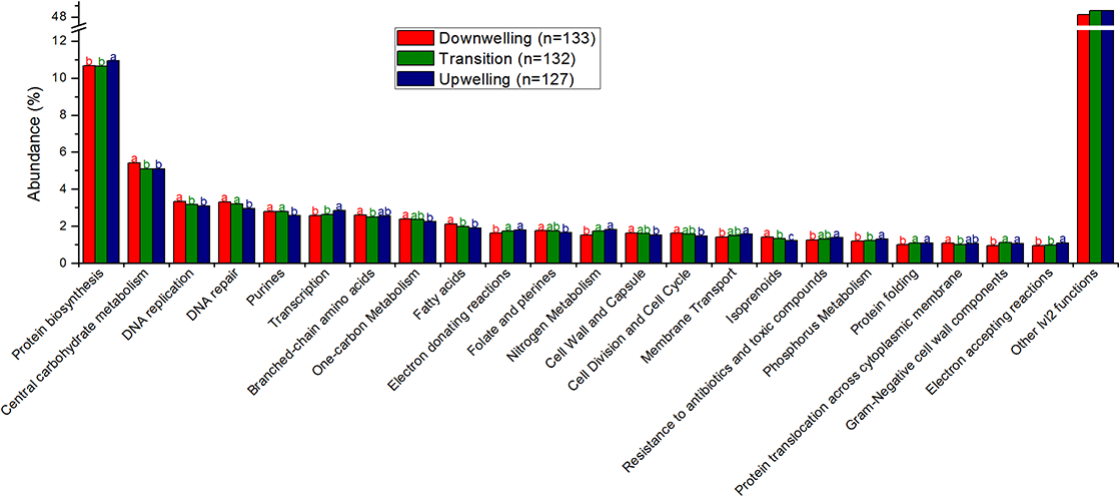
Significantly different most abundant cellular functions genes (Subsystems lvl2)–over 1% average abundance (relative percentage). The remaining functions are grouped as”Other lvl2 functions”. The total number of reads included in this analysis is 802,136. Different letters (a > b > c) represent statistically significant differences (corrected p <0.05), while “ab” are intermediate values.

The majority of cellular species identified were recognized as bacteria that are common members of the marine community, such as *Pelagibacter*, *Alteromonas* and several members of the *Flavobacteriales* and *Rhodobacteriales* orders (Fig 3). Except for *Pelagibacter* and *Alteromonas*, which increased with the upwelling, most bacteria seem more abundant in the downwelling period (Fig 3). Three exceptions to the bacterial dominance are noteworthy, having increased with the upwelling: *Nitrosopumilus maritimus*, a thaumarchaea; *Thalassiosira pseudonana*, a centric diatom; and *Ostreococcus lucimarinus*, a picoeukaryote (Fig 3, S2 Table). *Ostreococcus* and uncultivated *Gammaproteobacterium* HTCC 2207, in fact, peaked over the transition, suggesting that the transition benefited at least a few microbial species. S2 Table presents the detailed taxonomic annotation and statistical analysis, but all aspects highlighted in the text refer to statistically significant observations. The cellular functions annotated are more diverse than the viral functions, with protein biosynthesis, transcription, electron-accepting and electron-donating reactions (respiration), membrane transport (Ton/Tol, Zn, Mn), nitrogen metabolism (ammonia assimilation, denitrification, nitrogen fixation), phosphorus metabolism (phosphate), sulfate reduction, inorganic sulfur assimilation and protein folding responding positively to the upwelling (Fig 4, S3 Table for details). By contrast, most carbohydrate metabolism, DNA metabolism, cell division, membrane and cell wall synthesis and sulfur oxidation are more prevalent in downwelling conditions (Fig 4). Only branched chain amino acid metabolism (isoleucine degradation) was affected by the transition, and it was affected negatively. S3 Table presents the detailed functional annotation and statistical analysis, but all aspects highlighted in the text refer to statistically significant observations.

## Discussion

Despite the wind direction and water temperature indications of the upwelling being established, both microbial and chemical analysis suggest otherwise. From these viewpoints, the dynamics of a rising upwelling event was captured, including its transition period. On January 18^th^, the higher viral and microbial counts (including cyanobacteria) and higher ammonia levels point to the aftereffects of a downwelling condition, with a microbial food web and nitrogen cycling. This observation implies that the entrance of the upwelling occasionally takes longer to impact surface waters and the microbial plankton. On January 19^th^, the nitrate and phosphate levels rose, while the microbes diminished, representing a transition period uncoupled from the change in wind direction and delayed by three days in relation to it. On January 20^th^, the sustained higher levels of nitrate and phosphate plus increased silicate, probably biogenic from diatoms and/or upwelled from the bottom, suggest the establishment of the upwelling, which was also three days delayed in relation to the direction of the winds. This scenario is in accord with previous studies in this region [2, 4, 7, 10]. Therefore, samples from both femto- (virus) and pico/nanoplankton (bacteria, archaea and smaller eukaryotes) fractions were studied during this transition. While larger phytoplankton (e.g., diatoms) are clearly more prominent as primary producers in upwelling conditions [22, 23], the roles of picoplankton have been highlighted more recently [7, 24], particularly during the downwelling, where the microbial loop should be relevant [8]. It is arguable that the observable changes are a microbial community response or a change in water masses in response to the winds. In both cases, the following description of the pico- and femtoplankton still applies because, despite the direct cause, the changes are noted in surface waters during this transition. The gradual changes observed in the majority of the taxonomic and functional profiles (presented in full in the supplementary material) suggest that both causes may play a role.

### Downwelling conditions

Although the observation of prochlorococcal phages is unusual because their hosts are not commonly found in coastal sites as *Synechococcus* [25] (and indeed, in this study, only the latter were detected by auto-fluorescence in flow cytometry, as depicted in Table 2), these viruses could have drifted towards the coast as a result of the surface currents prevalent in these downwelling conditions, where these genera could flourish, or more likely could be misidentified, as cross-infection between these cyanobacterial genera is not uncommon [26, 27]. Both alternatives suggest a higher pressure of the identifiable viral loop over the bacterial primary producers in this downwelling condition, more akin to surface waters [28, 29]. Several alleged auxiliary metabolic genes (AMGs) [30] could be identified, as defined in the Results (Metagenomic characterization of viruses), and most related to host processes abundant during upwelling, suggesting an unpaired coupling, with viral genes adapted to conditions from a few days before. Most favored microbial cells in the downwelling conditions belong to the *Flavobacteriales* and the *Rhodobacteriales* orders, and a metabolic analysis of their sequences suggest their involvement in central carbohydrate metabolism and isoprenoid synthesis, among other processes. These orders were found to be positively correlated with temperature and negatively correlated with phosphate and nitrate in a previous upwelling growth rate assessment [31], which are conditions compatible with downwelling. The prevalence of type II fatty acid synthases, specific to bacteria and archaea [32], also corroborate the prevalence of prokaryotes during downwelling. Most nitrogen metabolism is reduced relative to upwelling conditions, but ammonia assimilation is comparatively (to other nitrogen metabolism varieties) high in this condition as well, hinting at microbial loop recycling. Despite higher chlorophyll contents, most oxygenic photosynthesis is reduced in downwelling conditions, but the sulfur oxidation pathways and type II photosystem proteins are elevated, suggesting a role for aerobic anoxygenic purple bacteria [33], which concurs with the abundance of ammonia and *Rhodobacteriales* noted above.

### Upwelling conditions

The upwelling conditions favor a different set of viruses mainly related to phages described previously in the deep chlorophyll maximum (DCM) of the Mediterranean [34], *Roseobacter* phages and algal viruses infecting *Bathycoccus* and *Chlorella*, shifting the recognizable pressure of the viral loop to unknown hosts (DCM), heterotrophic bacteria and eukaryotic algae. The alleged AMGs in these conditions are also reflected by the host metabolisms abundant during downwelling conditions, also hinting at an unpaired coupling with a lag of one to two days, between genes transferred by viruses and functions useful for hosts. These transduction suggestions should be tested properly in the future. A clear rise in diatom sequences is observable, as expected, which is interesting because eukaryal sequences are usually shadowed by the more abundant prokaryotic hosts in metagenomes and are even host associated. This suggests an impressive amount of these organisms, particularly considering that pre-filtration in 100 μm excluded larger cells. The rise in type I fatty acid synthases (eukaryal) corroborates this predominance [32]. The upwelling seems to counter-select all microbes except for *Pelagibacter*, *Alteromonas* and *Nitrosopumilus*, perhaps due to temperature reduction, as previously shown [2], at least during the initial stages captured here. Alternatively, the deep water mass could be supplying these microorganisms. *Pelagibacter ubique* is the most abundant bacteria in the oceans [35] and has been shown to thrive in upwelling environments as well [24]. In fact, increased membrane transport proteins, common for this group [5, 24], were found to be more abundant in these upwelling conditions. In the downwelling/upwelling transition, the growth of larger phytoplankton and their consumption of most nutrients could create a temporarily oligotrophic environment in which *P*. *ubique* would thrive. *Alteromonas macleodii* is known to present deep ecotypes [36], and together with *Nitrosopumilus maritimus* is likely being brought to the surface with the deep upwelling water masses [37], suggesting the possibility for easier sampling of these deep water genera in this region in upwelling conditions. The increasing concentration of nutrients in (temporarily) oligotrophic waters provides a suggested optimal environment for the opportunistic *A*. *macleodii* [36]. *Nitrosopumilus maritimus* growth could be stimulated by the increase in the ammonia concentration detected in downwelling conditions because this thaumarchaea is an autotrophic ammonia-oxidizing microbe [38]. It is possible that this group contributes to the so-called “priming” of the waters prior to larger phytoplankton growth [8] through conversion of the prevalent nitrogen form during downwelling (ammonia) to a more accessible nitrogen form for primary producers. Alternatively, this may represent one of many enriched nitrogen metabolism pathways in upwelling conditions, which would suggest a more complex scenario than algae growing on upwelled nitrates. In fact, we found that upwelling conditions corresponded to increases in several pathways of nitrate reduction (denitrification, nitrite reduction, nitrite and nitrate ammonification), coupled with ammonia assimilation, and even nitrogen fixation, suggesting that the already known importance of this nitrogen in this context [2] may be even wider than that of nitrates and also include some ammonia transformations. Sulfur seems to play a minor role in upwelling, though some increase in sulfur reduction is noted. It is likely that anaerobic metabolisms such as the several mentioned above, i.e., sulfur reduction and denitrification, could occur in the water column attached to particles [39]. Sediments (silicate) were suggested to arise with upwelled waters [4], and silicate is known to limit diatom productivity in some regions [40].

### Transition period

It was also possible to note some unusual behaviors during the transition between downwelling and upwelling. This fleeting niche favored some species, particularly the gammaproteobacterium HTCC2207 and the picoeukaryote *Ostreococcus lucimarinus*, suggesting that the mixture of surface waters of downwelling with cold deep upwelling waters presents conditions that favor some organisms. In fact, a similar trend for the abundance of *Ostreococcus* was noted in the front of an upwelling field in northern California, where upwelling and surface waters likely mix [41]. HTCC2207 has been implicated with high motility, phosphate metabolism and TonB transporters [42] although our analysis showed no increase in these metabolisms during the transition stage. The taxonomic changes in the viral community were more diverse, and viral species infecting the same host behaved differently, suggesting that the hosts may be more strongly affected and that the indirect effect on their viruses is less predictable. Additionally, a relative reduction in alleged viral AMGs was observed, with a predominance of viral structural components and viral nucleosides and nucleotides metabolism, which are basic viral functions. It is likely that the transient conditions hinder viruses adapted to either extreme, perhaps due to its brevity. Finally, it is worth noting that fewer changes were statistically significant in the transition period, either positively or negatively, and most annotations were significantly associated with either the downwelling or upwelling state, again corroborating the ephemeral state of the transition.

**Fig 5:**
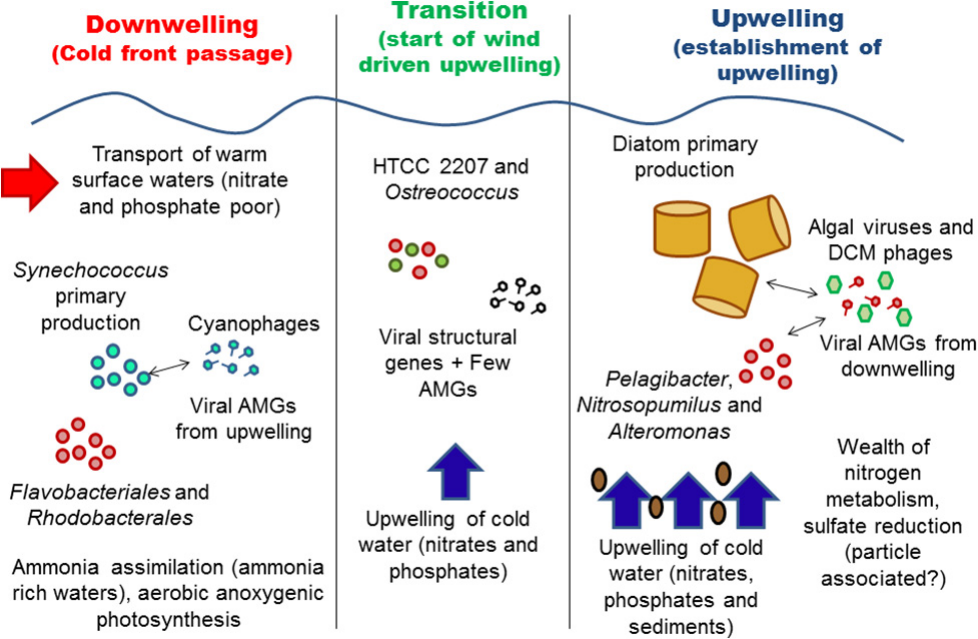
Conceptual map of upwelling dynamics. Main observations from this study as a proposal for structure of viral and bacterial communities (both in terms of organisms and functions) during the three stages analyzed here.

In summary, we obtained an overview of the functioning of the upwelling system (Fig 5) and provided a complete taxonomic and functional description of both femto- and picoplankton communities. The results have provided greater insight into the dynamics of the downwelling upwelling transition, suggesting that a wealth of metabolic processes and ecological interactions occur in the minute fractions of the plankton (femto, pico, nano). Although most of these microbial processes do not directly influence the traditional phytoplankton-zooplankton-fish food web, which confers ecological and economic importance to upwelling regions, several microbial processes can potentially impact higher trophic levels. These processes include aerobic anoxygenic photosynthesis in downwelling conditions and several nitrogen transformation pathways during upwelling, highlighting a role for ammonia and furthering our understanding of this ecosystem.

## Supporting Information

S1 Fig Wind direction data for January 2012, kindly provided by the Meteorological Data Storage Section (SADMET) of the Brazilian National Meteorological Institute (INMET). Directions corresponding to downwelling and upwelling are highlighted. Green arrows represent sampling days (18^th^, 19^th^ and 20^th^).
(TIF)

S1 Table Metagenomic technical data. (XLSX)

S2 Table Full taxonomic table split into separate tabs by analysis level. Tables contain the relative frequency employed in the statistical comparisons, p-values and effect sizes for each pairwise comparison, and a schematic interpretation of the statistically significant results (a>b>c).
(XLSX)

S3 Table Full functional table split into separate tabs by analysis level. Tables contain the relative frequency employed in the statistical comparisons, p-values and effect sizes for each pairwise comparison, and schematic interpretation of the statistically significant results (a>b>c).
(XLSX)
