# Supplementary figures and images for: Insights into the Microbial and Viral Dynamics of a Coastal Downwelling-Upwelling Transition
Source: PLoS One. 2015 Sep 1;10(9):e0137090. doi: 10.1371/journal.pone.0137090 (PMC4556663; doi:10.1371/journal.pone.0137090)

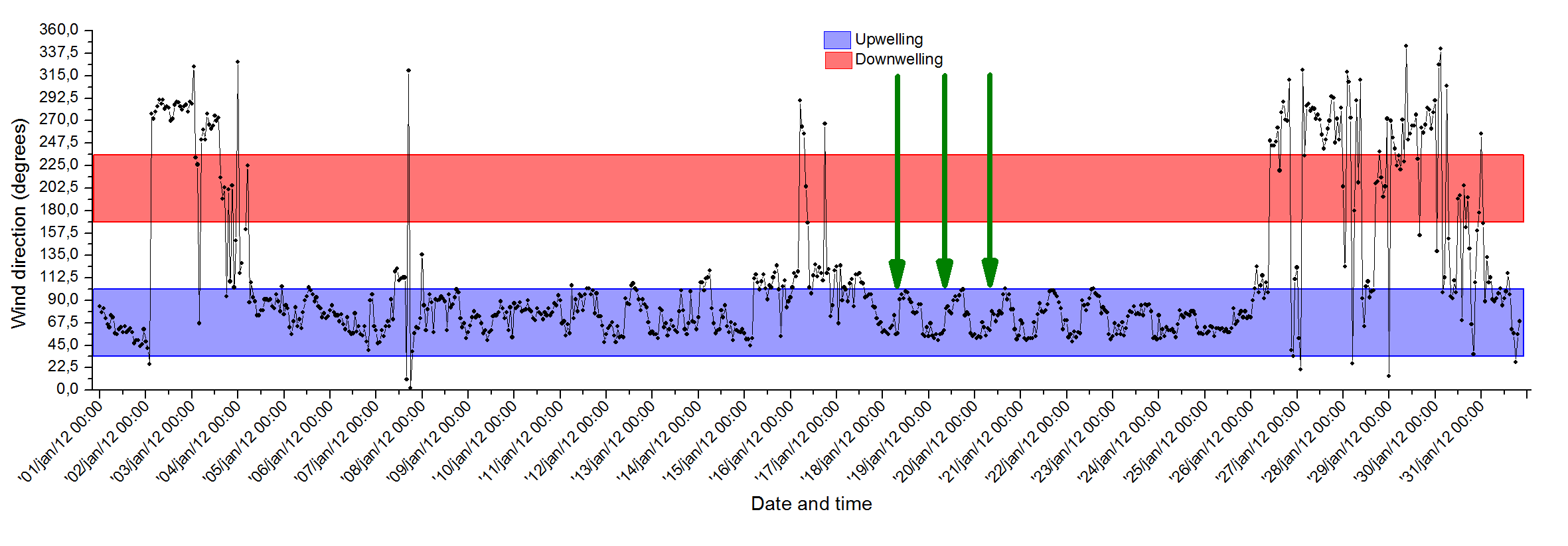

Supplement: S1 Fig — Directions corresponding to downwelling and upwelling are highlighted. Green arrows represent sampling days (18th, 19th and 20th). (TIF) [file pone.0137090.s001.tif]
